# Re-evaluation of the efficacy and safety of anti-Aβ monoclonal antibodies (lecanemab/donanemab) in the treatment of early Alzheimer’s disease

**DOI:** 10.3389/fphar.2025.1599048

**Published:** 2025-04-16

**Authors:** Haoyang Wang, Junying Pan, Mingming Zhang, Zengde Tan

**Affiliations:** Department of Tuina, First Affiliated Hospital, Heilongjiang University of Chinese Medicine, Harbin, Heilongjiang, China

**Keywords:** Aβ monoclonal antibody, lecanemab, donanemab, alzheimer’s disease, clinical outcomes, adverse reactions

## Abstract

**Objective:**

To systematically evaluate the efficacy and safety of anti-Aβ monoclonal antibodies (Lecanemab/Donanemab) in the treatment of early Alzheimer’s disease (AD) and to provide evidence for rational clinical use.

**Methods:**

We searched databases including PubMed, Embase, Cochrane, Web of Science, CNKI, and the Chinese Biomedical Literature Database for relevant literature on the use of anti-Aβ monoclonal antibodies in treating early AD. Two reviewers independently screened the literature, extracted data, and conducted meta-analysis using RevMan 5.4.

**Results:**

A total of five clinical studies were included. Meta-analysis results showed that in terms of clinical outcomes, Lecanemab/Donanemab outperformed the control group in ADCOMS, CDR-SB, ADAS-Cog 14, and amyloid burden on PET. Regarding safety, the relative risk of amyloid-related imaging abnormalities (ARIA) in patients treated with Lecanemab/Donanemab was 4.35 times higher than the control group, with significantly higher risks of ARIA-E and ARIA-H. Among other adverse events, the risk of superficial siderosis of the central nervous system was notably higher and statistically significant.

**Conclusion:**

Lecanemab/Donanemab can improve memory, cognitive function, and daily living abilities in patients with early AD, significantly reduce the composite score of Alzheimer’s disease, and inhibit the accumulation of amyloid peptides, thereby alleviating symptoms and improving the condition.

## 1 Introduction

Alzheimer’s disease (AD), the most common form of dementia, is characterized by progressive memory loss, cognitive dysfunction, language impairment, personality and behavioral changes, and declining orientation and judgment. In the final stages, patients lose all ability to care for themselves (Scheltens et al., 2021). Due to its high incidence and significant social impact, AD has become a major global public health challenge. The World Health Organization (WHO) predicts that the number of AD patients worldwide will reach 82 million by 2030 and 152 million by 2050 (Zhang et al., 2020). In China, there are currently six million AD patients, with the number expected to exceed 20 million by 2050. This alarming trend has prompted governments and researchers worldwide to accelerate related studies (Zhang et al., 2020).

The complex clinical manifestations of AD are multidimensional: in addition to core memory impairment, patients often experience aphasia (loss of language function), apraxia (impaired motor skills), agnosia (impaired object recognition), visuospatial deficits, executive dysfunction (impaired planning and decision-making), and significant personality and behavioral changes, ultimately leading to irreversible dementia or death (Weller and Budson, 2018). The pathological mechanisms are based on two hallmark changes: the deposition of neurotoxic amyloid-β (Aβ) plaques in the brain and neurofibrillary tangles (NFTs) caused by hyperphosphorylated tau protein, both of which contribute to neuronal apoptosis and synaptic dysfunction (Mantzavinos and Alexiou, 2017).

Currently, the main pharmacological treatments for AD (Knapskog et al., 2021; Briggs et al., 2016) are cholinesterase inhibitors (ChEIs), including donepezil, rivastigmine, and galantamine, which increase acetylcholine levels in the synaptic cleft and are used to treat mild to moderate AD. These drugs can improve cognitive function, overall impression, daily living abilities, and psychiatric symptoms. Another drug, memantine, an NMDA receptor antagonist, is approved for moderate to severe dementia and can help manage delusions and agitation in patients with moderate to severe AD (Briggs et al., 2016). Other drugs, such as huperzine A and oxiracetam, are also used in AD treatment (Briggs et al., 2016). However, existing drugs only partially alleviate symptoms or slow disease progression, and there is no treatment that can reverse the pathological process.

In recent years, despite the potential shown by Aβ and tau-targeted therapies in animal studies, several international Phase III clinical trials (e.g., Aducanumab, Semorinemab) have failed to meet their endpoints, highlighting the complexity of AD drug development and the challenges of clinical translation (Rostagno, 2022). In this context, disease-modifying therapies (DMT) targeting Aβ, such as monoclonal antibodies, have emerged as a new direction (Narang et al., 2020). Researchers believe that Aβ metabolic imbalance and its neurotoxicity are central to AD pathology, with abnormal aggregation directly damaging neurons and causing cognitive impairment (Ono, 2018; Shi et al., 2022). In January 2023, the anti-Aβ protofibril antibody lecanemab was approved by the FDA for the treatment of early AD, followed by donanemab in July 2024, which significantly delayed cognitive decline and disease progression (Cummings et al., 2023; Kurkinen, 2023). Although both drugs have shown positive results in clinical trials, the association between amyloid-related imaging abnormalities and the APOE ε4 genotype remains controversial. This study aims to integrate all Phase III trial data post-2021 to re-evaluate the clinical efficacy and safety of lecanemab/donanemab, providing more evidence-based decision-making for AD treatment.

## 2 Materials and methods

### 2.1 Inclusion and exclusion criteria

Inclusion criteria for this study were: 1) Published RCTs or clinical trials; 2) Participants were adults with early AD; 3) The intervention involved the use of Lecanemab or Donanemab in the experimental group and placebo in the control group, with treatment lasting at least 72 weeks; 4) Outcome measures included clinical outcomes (Clinical Dementia Rating Scale—Sum of Boxes (CDR-SB), Alzheimer’s Disease Assessment Scale (ADAS-Cog 14), Alzheimer’s Disease Composite Score (ADCOMS), and amyloid burden on PET), amyloid-related imaging abnormalities, and common adverse events (death, serious adverse events, falls, dizziness, headache, superficial siderosis of the central nervous system, arthralgia, urinary tract infection, diarrhea, anxiety).

Exclusion criteria were: 1) Literature lacking baseline data or other essential information; 2) Meta-analyses, case reports, reviews, or commentary articles; (3) Suspected duplicate publications.

### 2.2 Literature search strategy

We searched English and Chinese databases, including Cochrane Library, PubMed, Embase, Medline, Web of Science, VIP, CNKI, and Wanfang Data, for RCTs and clinical trials on Lecanemab or Donanemab in AD treatment, from inception to March 2025. Search terms included “Lecanemab,” “Donanemab,” “Amyloid-β plaques,” “Aβ plaques,” and “Alzheimer’s Disease.” A combination of subject headings and free-text terms was used, and authors were contacted for additional information when necessary.

### 2.3 Literature screening and quality assessment

Two researchers independently screened and assessed the literature, resolving disagreements through discussion. The risk of bias in the included studies was evaluated using the Cochrane Handbook’s risk of bias tool for RCTs (Gan et al., 2018). Extracted information included the title, first author, publication year, sample size, treatment methods, intervention duration, and outcome measures. Two other researchers verified the extracted data before analysis.

### 2.4 Statistical analysis

Meta-analysis was performed using RevMan 5.4 software. Dichotomous data were analyzed using odds ratios (OR) and relative risks (RR) with 95% confidence intervals (CI). Heterogeneity among studies was assessed using the χ^2^ test (α = 0.1), and the I^2^ statistic was used to quantify heterogeneity. If no statistical heterogeneity was found, a fixed-effects model was used; if heterogeneity was present, its source was analyzed, and a random-effects model was applied after excluding significant clinical heterogeneity. Significant clinical heterogeneity was addressed through subgroup or sensitivity analysis, or descriptive analysis only. Publication bias was assessed using funnel plots, as recommended by the Cochrane Handbook.

## 3 Results

### 3.1 Literature search and quality assessment results

**FIGURE 1:**
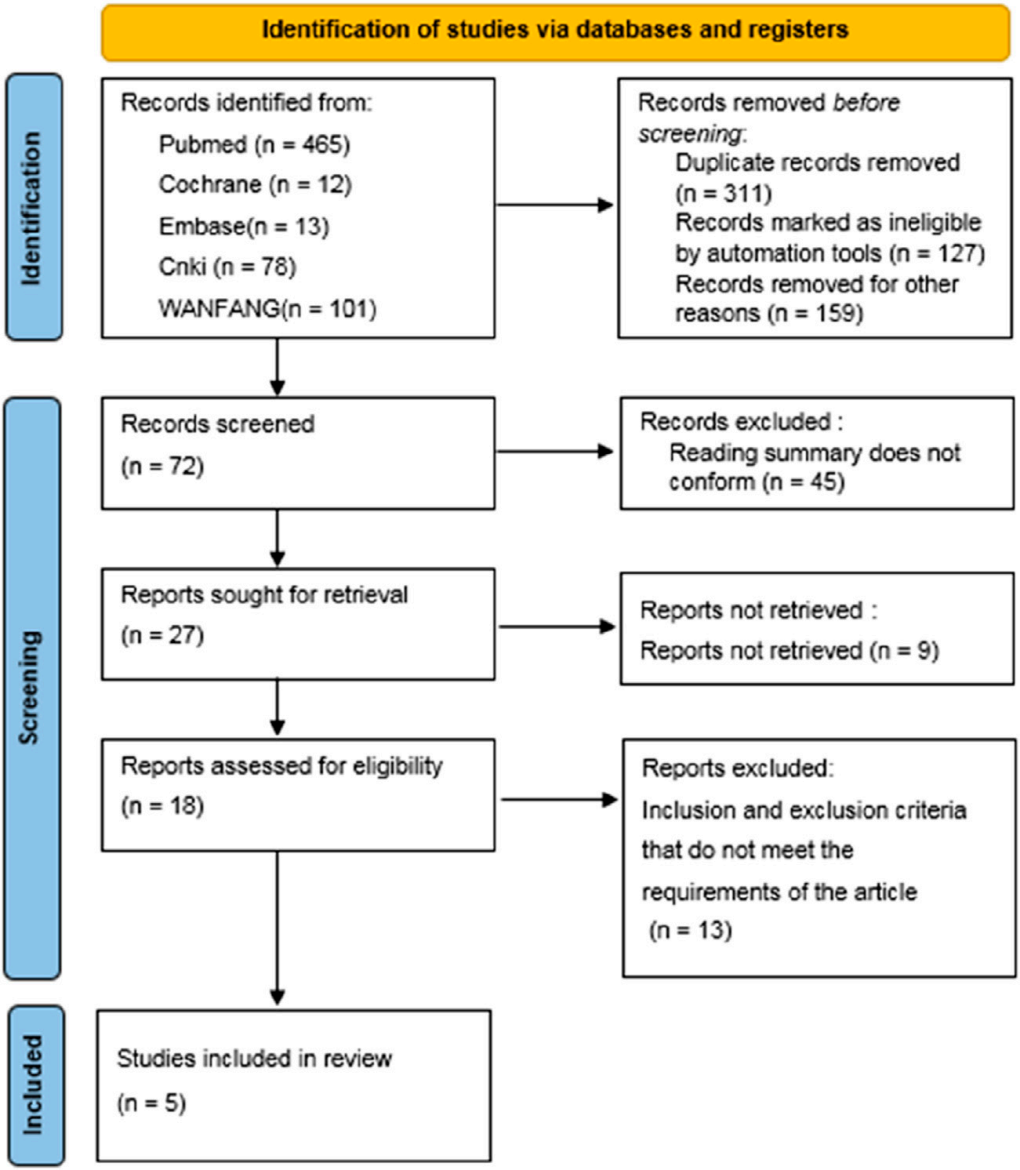
Flow chart of literature search.

A total of 669 relevant literatures were initially detected, 311 duplicate records were removed, 127 records were marked as unqualified by EndNote software, 159 records were deleted for other reasons, 45 reading abstracts did not meet the requirements, nine reports were not retrieved, and 13 did not meet the inclusion and exclusion criteria. Finally, a total of five literatures were included (Mintun et al., 2021; van Dyck et al., 2023; McDade et al., 2022; Swanson et al., 2021; Honig et al., 2024) (Figure 1).

### 3.2 Basic characteristics and quality assessment

**FIGURE 2:**
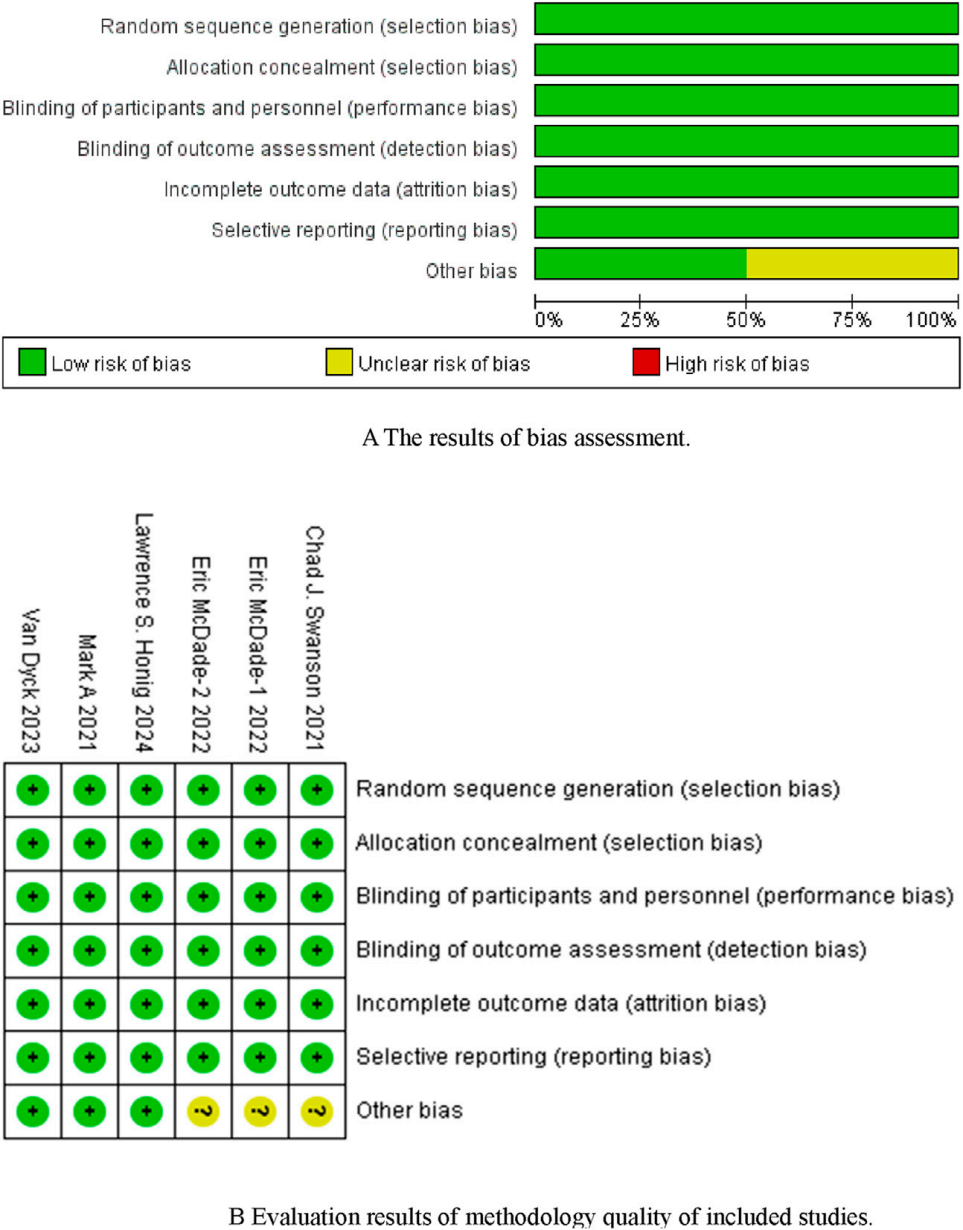
**(A)** The results of bias assessment. Figure **(B)** Evaluation results of methodology quality of included studies.

Five studies (Mintun et al., 2021; van Dyck et al., 2023; McDade et al., 2022; Swanson et al., 2021; Honig et al., 2024) were included, with a total sample size of 4,824 patients (2,410 in the experimental group and 2,414 in the control group). Table 1 shows the basic characteristics of the included studies, and Figures 2A, B presents the quality assessment results.

**TABLE 1:** Characteristics and methodological quality of involved studies (Mintun et al., 2021; van Dyck et al., 2023; McDade et al., 2022; Swanson et al., 2021; Honig et al., 2024).

Author	Year	Group	Intervention measures	No.	Female sex	Age	APOE ε4 carrier
Mark A. (Mintun et al., 2021)	2021	EXP	Donanemab	131	68	75.0 ± 5.6	95
		CON	placebo	126	65	75.4 ± 5.4	92
van Dyck (van Dyck et al., 2023)	2023	EXP	Lecanemab	859	443	71.4 ± 7.9	592
		CON	placebo	875	464	71.0 ± 7.8	600
Eric McDade-1 (McDade et al., 2022)	2022	EXP	Lecanemab	30	---	71.3 ± 7.5	---
		CON	placebo	40	---	71.1 ± 8.9	---
Eric McDade-2 (McDade et al., 2022)	2022	EXP	Lecanemab	246	110	71.3 ± 7.5	218
		CON	placebo	238	137	71.1 ± 8.9	169
Chad J. Swanson (Swanson et al., 2021)	2021	EXP	Lecanemab	246	110	71 (53–90)	218
		CON	placebo	238	137	72 (50–89)	169
Lawrence S. Honig (Honig et al., 2024)	2024	EXP	Lecanemab	898	462	71.4 ± 7.9	620
		CON	placebo	897	476	71.1 ± 7.8	611

### 3.3 Meta-analysis results

#### 3.3.1 Clinical outcomes

Three studies reported clinical outcomes (ADCOMS, CDR-SB, ADAS-Cog 14, amyloid burden on PET). Meta-analysis results showed: (1) ADCOMS: No significant heterogeneity among studies (I^2^ = 0%, P = 0.76), and a fixed-effects model was used. Lecanemab/Donanemab significantly reduced the Alzheimer’s Disease Composite Score compared to the control group [HR = −0.05, 95% CI (−0.07, −0.03), P < 0.00001]; (2) CDR-SB: No significant heterogeneity (I^2^ = 0%, P = 0.55), and a fixed-effects model was used. Lecanemab/Donanemab significantly improved memory, cognitive function, and daily living abilities compared to the control group [HR = −0.49, 95% CI (−0.67, −0.30), P < 0.00001]; (3) ADAS-Cog 14: No significant heterogeneity (I^2^ = 47%, P = 0.15), and a fixed-effects model was used. Lecanemab/Donanemab significantly reduced cognitive impairment compared to the control group [HR = −1.06, 95% CI (−1.54, −0.57), P < 0.0001]; (4) Amyloid burden on PET: Significant heterogeneity (I^2^ = 95%, P < 0.00001), and a random-effects model was used. Lecanemab/Donanemab significantly reduced amyloid deposition compared to the control group [HR = −72.99, 95% CI (−88.58, −57.41), P < 0.00001] (Table 2).

**TABLE 2:** Meta-analysis results of Clinical Outcomes.

Outcomes	Studies	Sample size		Heterogeneity		Effect model	Meta analysis		
		Donanemab/Lecanemab	Placebo	*I* ^*2*^/%	*P*		HR	95%*CI*	*P*
ADCOMS	3	1,381	1,391	0	0.76	Fixed	−0.05	−0.07, −0.03	<0.00001
CDR-SB	3	1,381	1,391	0	0.55	Fixed	−0.49	−0.67, −0.30	<0.00001
ADAS-Cog 14	3	1,135	1,153	47	0.15	Fixed	−1.06	−1.54, −0.57	<0.0001
Amyloid burden on PET	2	1,135	1,153	95	<0.00001	Random	−72.99	−88.58, −57.41	<0.00001

In summary, Lecanemab/Donanemab can improve memory, cognitive function, and daily living abilities in patients with early AD, significantly reduce the Alzheimer’s Disease Composite Score, and inhibit amyloid peptide accumulation, thereby alleviating symptoms and improving the condition.

#### 3.3.2 Amyloid-related imaging abnormalities (ARIA)

##### 3.3.2.1 ARIA-E and ARIA-H

**FIGURE 3:**
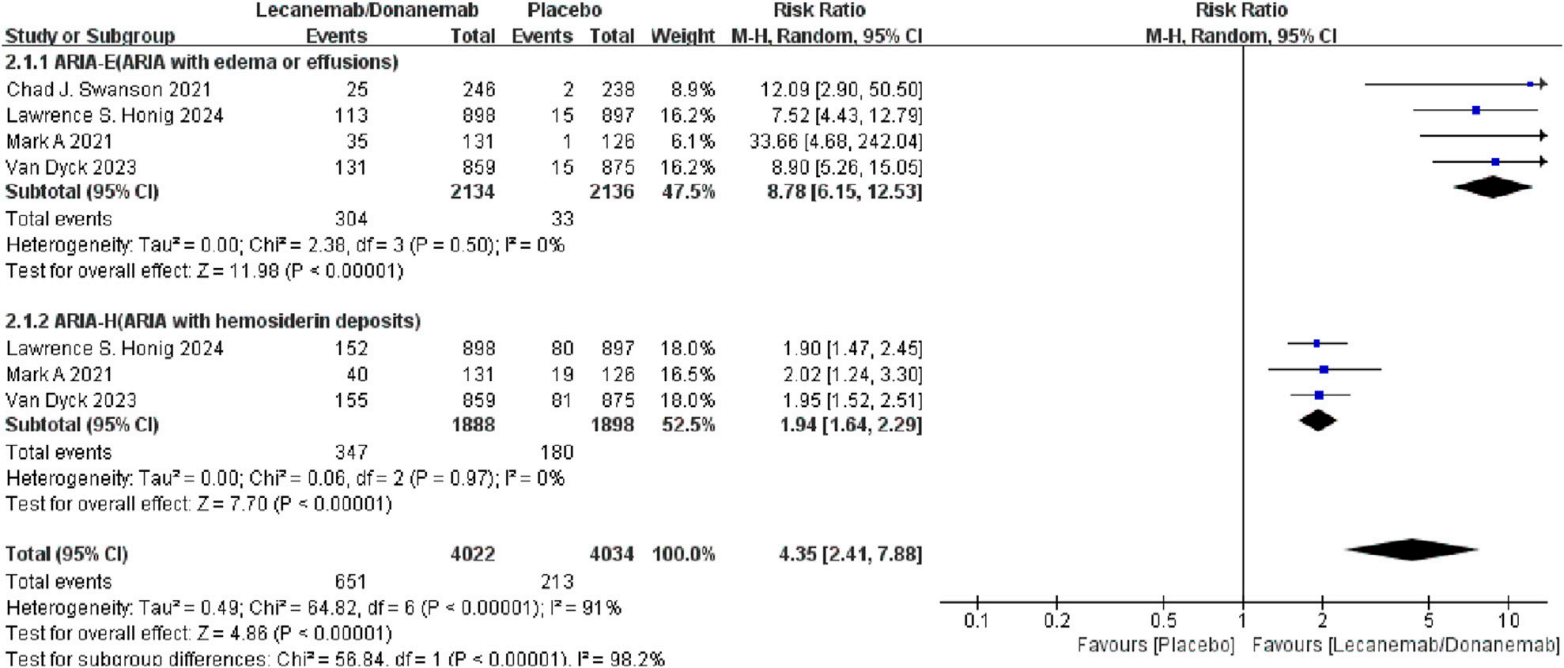
Meta-analysis results of Amyloid-related imaging abnormalities.

Amyloid-related imaging abnormalities (ARIA) include ARIA-E (edema or effusions) and ARIA-H (hemosiderin deposits). Four studies reported ARIA-E, and three studies reported ARIA-H. Meta-analysis results showed that the relative risk of ARIA in patients treated with Lecanemab/Donanemab was 4.35 times higher than the control group [RR = 4.35, 95% CI (2.41, 7.88), P < 0.00001]. Subgroup analysis showed that Lecanemab/Donanemab significantly increased the risk of ARIA-E [RR = 8.78, 95% CI (6.15, 12.53), P < 0.00001] and ARIA-H [RR = 1.94, 95% CI (1.64, 2.29), P < 0.00001] compared to the control group. These results are likely related to the drug’s mechanism of action, as both drugs clear Aβ from the brain (Lecanemab targets soluble protofibrils, while Donanemab targets deposited plaques), which may lead to rapid clearance of perivascular Aβ. In patients with cerebral amyloid angiopathy, the disruption of Aβ deposits in vessel walls may weaken vascular integrity, leading to leakage (ARIA-E) or hemorrhage (ARIA-H) (Figure 3).

##### 3.3.2.2 ARIA-E

**FIGURE 4:**
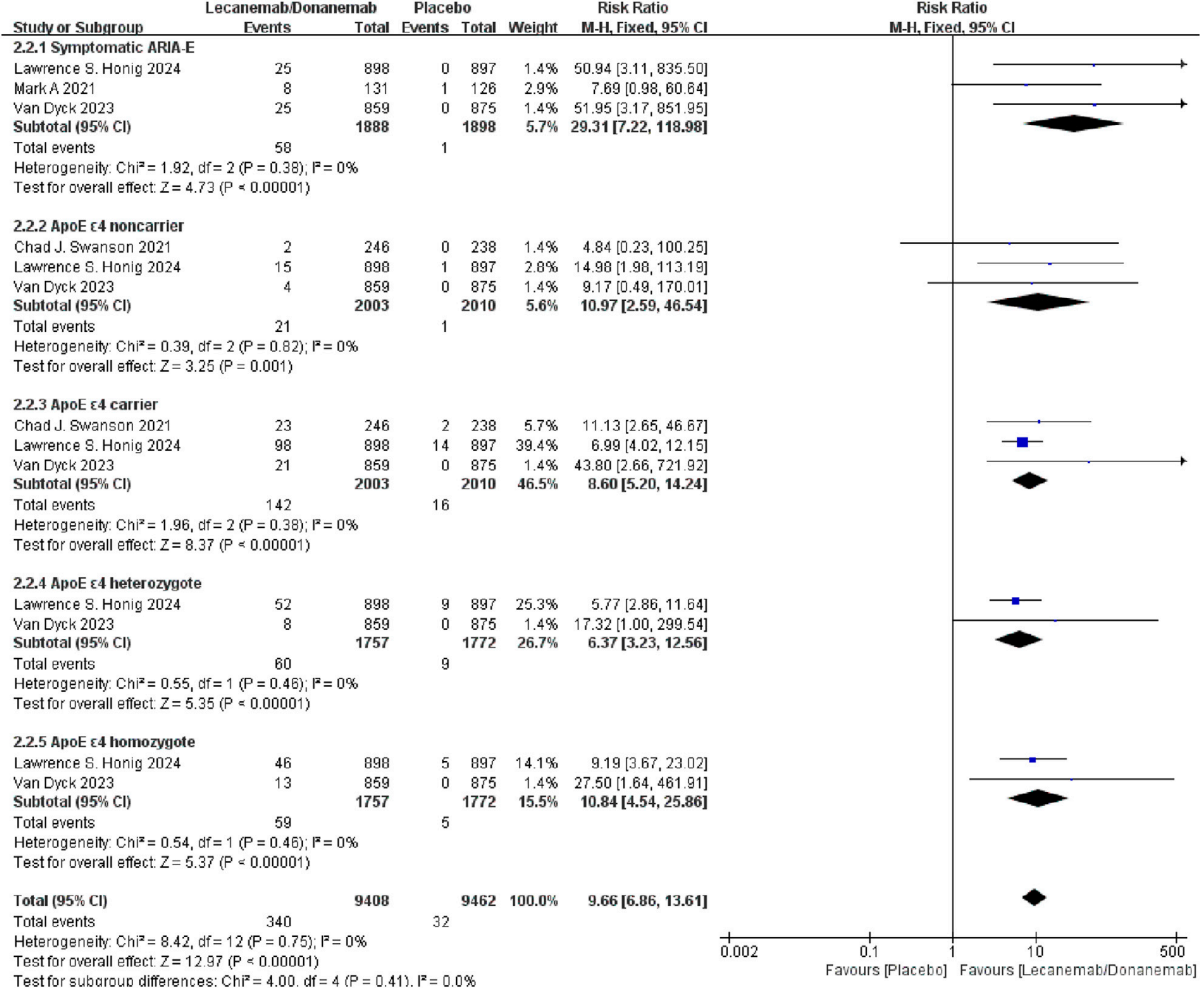
Meta-analysis results of ARIA with edema or effusions.

Subgroup analysis of ARIA-E (edema or effusions) included symptomatic ARIA-E, ApoE ε4 non-carriers, ApoE ε4 carriers, ApoE ε4 heterozygotes, and ApoE ε4 homozygotes. Meta-analysis results showed no significant heterogeneity among studies (I^2^ = 0%, P = 0.75), and a fixed-effects model was used. Subgroup analysis indicated that the risk of ARIA-E in ApoE ε4 carriers treated with Lecanemab/Donanemab was 10.97 times higher than the control group, while in non-carriers, the risk was 8.60 times higher. Additionally, the risk of ARIA-E in ApoE ε4 heterozygotes was 6.37 times higher, and in ApoE ε4 homozygotes, it was 10.84 times higher. These results suggest that ARIA-E incidence is associated with ApoE ε4, and the risk increases in carriers and homozygotes (Figure 4).

##### 3.3.2.3 ARIA-H

**FIGURE 5:**
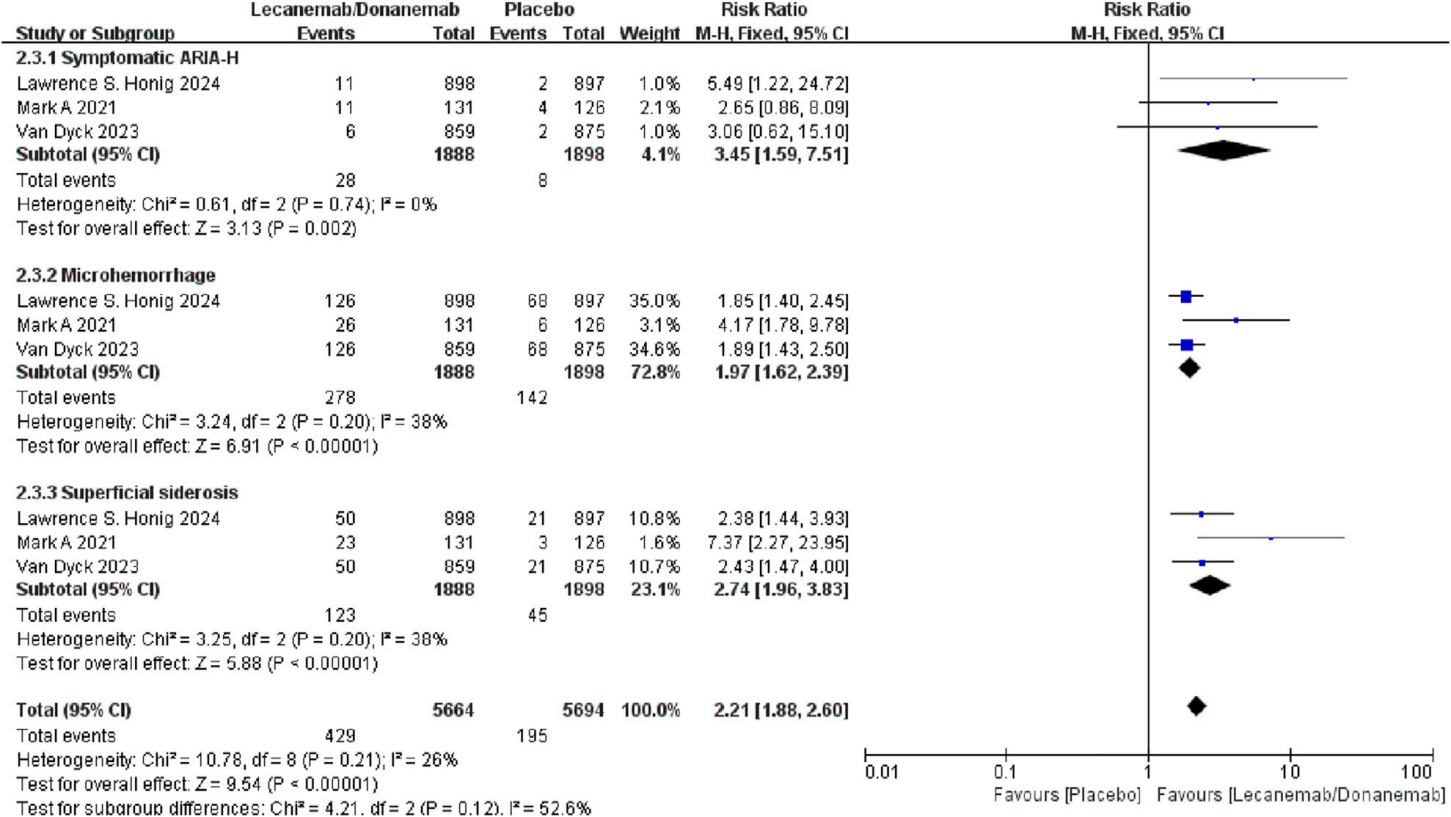
Meta-analysis results of ARIA with hemosiderin deposits.

Meta-analysis results showed that the relative risk of ARIA-H (hemosiderin deposits) in patients treated with Lecanemab/Donanemab was 2.21 times higher than the control group [RR = 2.21, 95% CI (1.88, 2.60), P < 0.00001]. Subgroup analysis showed that patients treated with Lecanemab/Donanemab had an increased risk of microhemorrhage [RR = 1.97, 95% CI (1.62, 2.39), P < 0.00001] and superficial siderosis [RR = 2.74, 95% CI (1.96, 3.83), P < 0.00001], with superficial siderosis having a higher risk (Figure 5).

#### 3.3.3 Other adverse events

**FIGURE 6:**
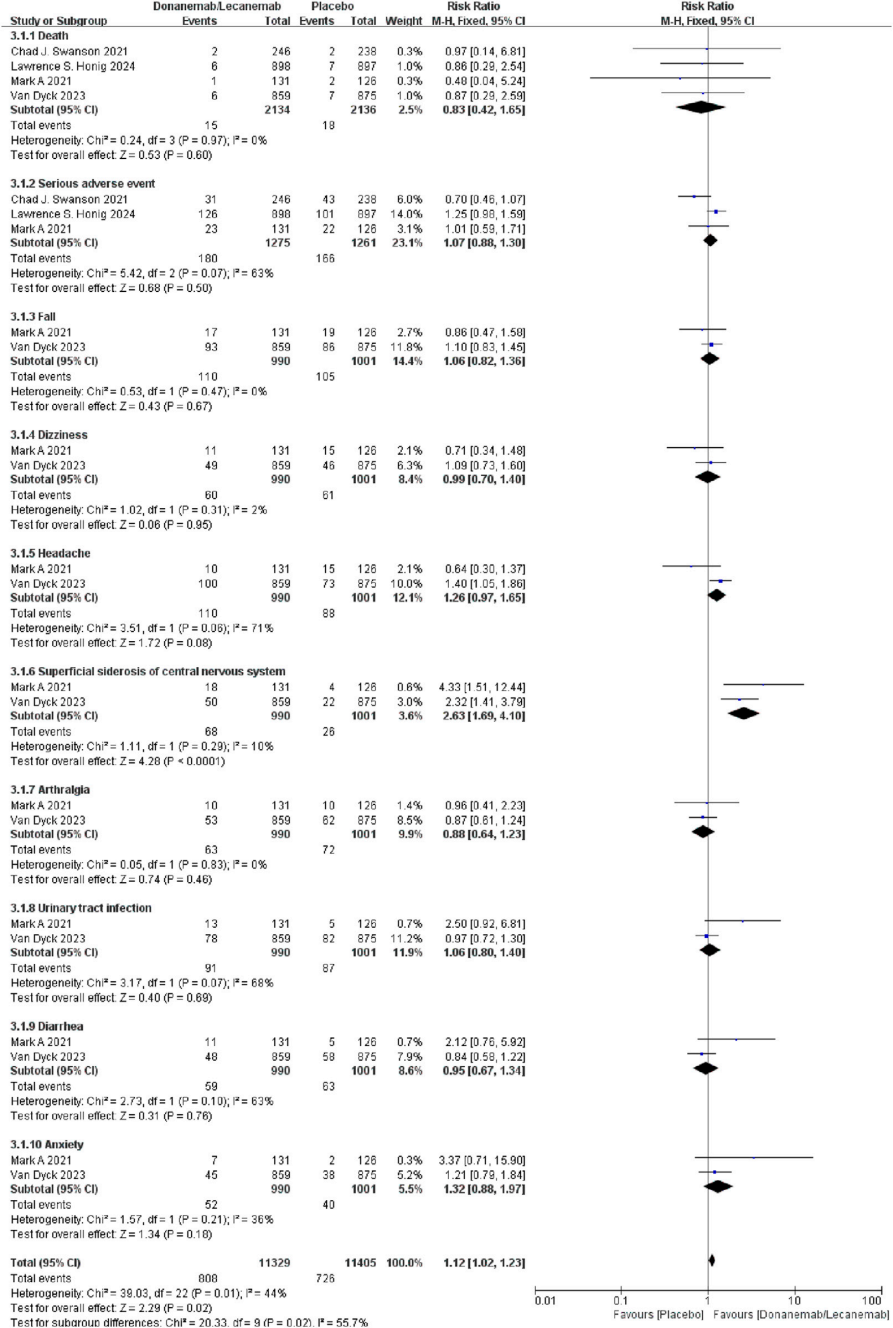
Meta-analysis results of other adverse events.

Meta-analysis results showed that the relative risk of other adverse events in patients treated with Lecanemab/Donanemab was 1.12 times higher than the control group [RR = 1.12, 95% CI (1.02, 1.23), P = 0.02]. Subgroup analysis showed no significant difference in the risk of death, serious adverse events, falls, dizziness, headache, arthralgia, urinary tract infection, diarrhea, or anxiety between the treatment and control groups. However, the risk of superficial siderosis of the central nervous system was 2.63 times higher in the treatment group [RR = 2.63, 95% CI (1.69, 4.10), P < 0.0001]. This may be due to Aβ antibodies triggering local inflammatory responses (e.g., complement activation or cytokine release), increasing blood-brain barrier permeability and allowing blood components (including red blood cells) to leak into brain tissue. Chronic leakage leads to iron deposition as the brain’s clearance capacity is exceeded (Figure 6).

### 3.4 Publication bias analysis

**FIGURE 7:**
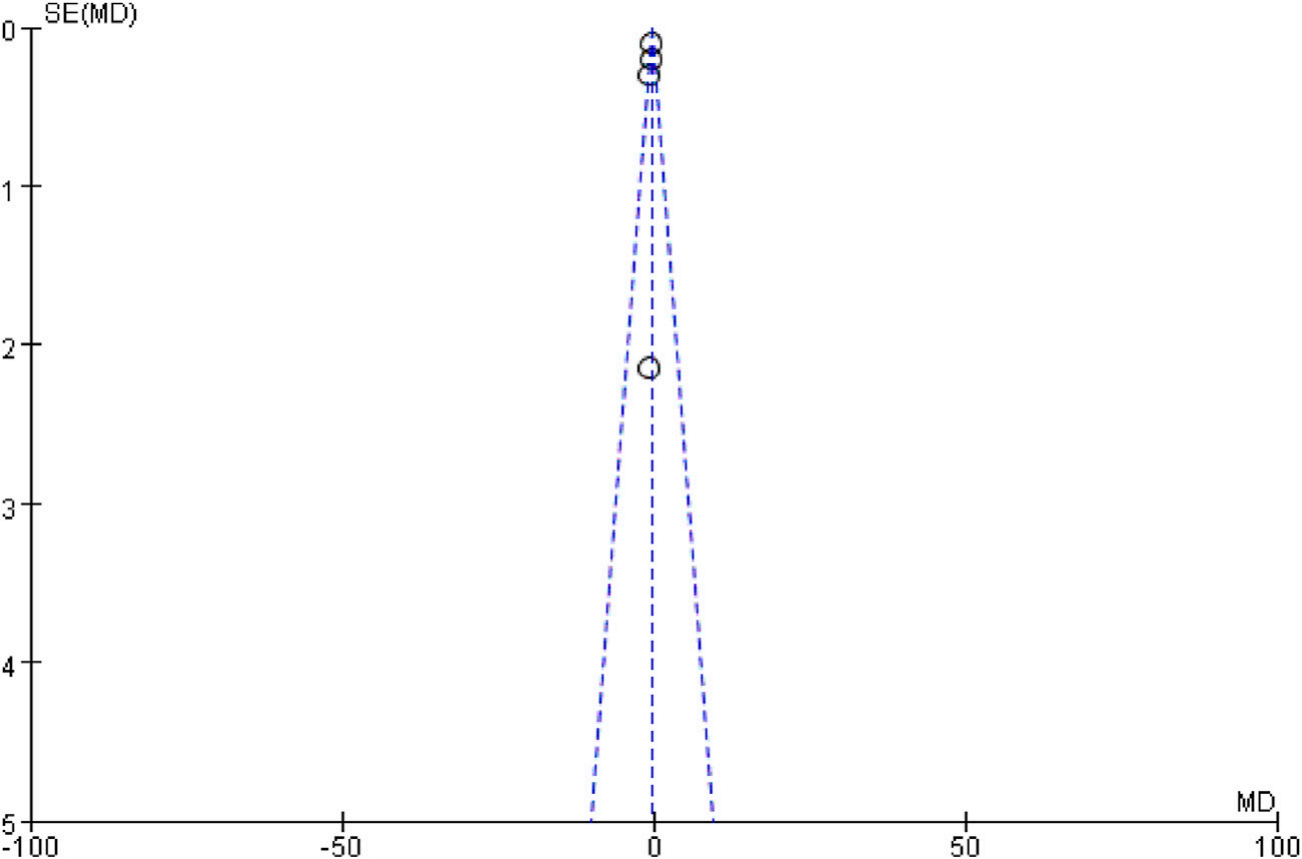
Funnel diagram of CDR-SB.

Funnel plots were used to assess publication bias, as recommended by the Cochrane Handbook. CDR-SB was selected as the indicator for publication bias, and the funnel plot showed a symmetrical distribution, suggesting no significant publication bias (Figure 7).

## 4 Discussion

Alzheimer’s disease (AD) is a neurodegenerative disorder characterized by progressive cognitive dysfunction and is the leading cause of neurocognitive disorders (NCD) in the elderly (Zheng and Wang, 2025). Its typical clinical manifestations include progressive memory decline (with anterograde and retrograde amnesia being prominent), executive dysfunction, and loss of daily living abilities. The incidence and prevalence of AD increase exponentially with age. The hallmark neuropathological features of AD include extracellular amyloid-β (Aβ) plaques and intracellular neurofibrillary tangles caused by hyperphosphorylated tau protein. Research has confirmed that the Aβ cascade plays a central role in AD pathogenesis, with abnormal aggregation triggering neuronal toxicity, synaptic dysfunction, and neuroinflammation (Delport and Hewer, 2022).

Current clinical management of AD primarily involves multimodal intervention strategies. Traditional pharmacological treatments include two main classes: cholinesterase inhibitors (e.g., donepezil), which enhance cholinergic neurotransmission to improve symptoms, and NMDA receptor antagonists (e.g., memantine), which modulate the glutamatergic system to provide neuroprotection. However, these drugs only temporarily alleviate symptoms and do not slow disease progression. In terms of adjunctive therapies, acupuncture (including conventional needling and electroacupuncture at specific points) and massage techniques are often used to improve cognitive function, potentially by modulating cerebral blood flow and neuroplasticity (Zhang and Chuxiao, 2022).

With advances in understanding AD pathology, disease-modifying therapies (DMT) targeting Aβ have become a focus of research. Current evidence suggests that the imbalance between Aβ production and clearance, leading to abnormal brain deposition, is a key initiating factor in the AD pathological cascade. Aβ oligomers can induce synaptic toxicity, mitochondrial dysfunction, and blood-brain barrier damage, ultimately leading to neuronal loss and cognitive impairment (Walsh and Teplow, 2012; Regland and Gottfries, 1992; Nakano et al., 2022). In this context, the new anti-Aβ monoclonal antibodies Lecanemab and Donanemab have shown significant therapeutic potential.

Lecanemab, a humanized IgG1 monoclonal antibody, exerts its therapeutic effects by specifically binding to soluble Aβ oligomers and protofibrils. Animal studies have shown that Lecanemab significantly reduces the number of pathogenic Aβ plaques in AD models, inhibits Aβ aggregation, and selectively clears Aβ protofibrils from brain tissue and cerebrospinal fluid (Shi et al., 2022). Population pharmacokinetic studies indicate that the drug follows a linear two-compartment model: compared to a 10 mg/kg monthly dosing regimen, biweekly dosing more rapidly reduces amyloid PET standardized uptake value ratios (SUVr) and plasma p-tau181 levels, while significantly increasing the Aβ42/40 ratio. Notably, the half-life of amyloid re-accumulation in the brain after treatment cessation is up to 4 years, while plasma biomarkers (Aβ42/40 and p-tau181) recover much faster than brain pathology (Hayato et al., 2022).

Donanemab, a recombinant humanized IgG1 monoclonal antibody developed by Eli Lilly, works by specifically recognizing and clearing deposited Aβ plaques. The drug was approved by the FDA in July 2024 for the treatment of early AD (mild NCD or mild dementia stages), making it the first Aβ-targeted therapy that does not require lifelong administration. Its innovative treatment strategy involves periodic dosing to achieve Aβ plaque clearance, and it has not yet entered the Chinese market (Kang, 2024).

This study systematically evaluated the clinical efficacy and biological effects of anti-Aβ monoclonal antibodies (Lecanemab/Donanemab) on patients with early AD through a meta-analysis. The results showed that the intervention group had significant advantages in core indicators such as the Alzheimer’s Disease Composite Score (ADCOMS), Clinical Dementia Rating-Sum of Boxes (CDR-SB), Alzheimer’s Disease Assessment Scale-Cognitive Subscale (ADAS-Cog 14), and amyloid PET load, indicating that these drugs have dual effects of improving cognitive function and clearing pathological proteins. In terms of ADCOMS score, the intervention group had a reduction of 0.05 units compared with the control group (95% CI: 0.07 to −0.03), which is consistent with the disease-modifying effect observed in the AHEAD 3–45 study (Raman et al., 2024). ADCOMS, as a composite endpoint integrating cognition, function, and biomarkers, may be improved due to the indirect regulatory effect of Aβ clearance on synaptic plasticity (DeVos et al., 2017). The CDR-SB score decreased by 0.49 units (95% CI: 0.67 to −0.30), especially in the dimension of self-care ability, suggesting that anti-Aβ treatment may delay the degenerative changes in functional brain regions (such as the default mode network) by reducing Aβ-mediated neuronal network damage (Singh et al., 2023). It is worth noting that the cognitive improvement amplitude shown by the ADAS-Cog 14 scale (SMD = −1.06) was significantly higher than that of traditional cholinesterase inhibitors (usually 0.3–0.5), which is in line with the efficacy trend of Lecanemab in the Clarity AD study (van Dyck et al., 2023), indicating that disease-modifying treatment may break through the therapeutic bottleneck of symptomatic treatment.

PET imaging analysis showed that the amyloid load in the intervention group decreased by 72.99 SUVr units (95% CI: 88.58 to −57.41), which may be related to the high-affinity clearance characteristics of Donanemab for mature plaques. However, there is a dissociation between amyloid clearance and the magnitude of cognitive improvement (e.g., only a 1.06 unit improvement in ADAS-Cog), a phenomenon also reported in the TRAILBLAZER-ALZ four trial (Mintun et al., 2021). This may reflect the following mechanisms: (1) Aβ clearance requires multiple steps, such as synaptic remodeling and neuroinflammatory relief, to be translated into clinical benefits; (2) The continuous progression of tau pathology may offset the potential benefits of Aβ clearance; (3) The existing scales are not sensitive enough to capture the subtle changes in early AD.

This study focused on the risk of amyloid-related imaging abnormalities (ARIA) and other adverse events. The results showed that anti-Aβ treatment significantly increased the risk of ARIA, with specific associations between its subtypes and genetic susceptibility, which has important guiding significance for clinical risk stratification and drug monitoring.

The occurrence of ARIA-E (edema/effusion) and ARIA-H (hemosiderin deposition) is closely related to the mechanism of drug clearance of intracerebral Aβ. Lecanemab targets soluble Aβ protofibrils, while Donanemab clears dense plaques. Both may accelerate the stripping of Aβ around blood vessels, leading to damage to the vascular basement membrane structure. This study found that anti-Aβ treatment increased the overall risk of ARIA by 4.35 times (RR = 4.35), with the most significant increase in the risk of ARIA-E (RR = 8.78). It is worth noting that the APOE ε4 genotype has a bidirectional regulatory effect on the risk of ARIA-E: non-carriers have a 10.97-fold increase in risk, while homozygous patients have a 10.84-fold increase in risk. This suggests that APOE ε4 may affect the occurrence of ARIA through a dual mechanism—both by exacerbating the severity of cerebral amyloid angiopathy (CAA) and by amplifying the blood-brain barrier disruption effect through apolipoprotein E-mediated inflammatory responses. Although the risk of ARIA-H (RR = 2.21) is lower than that of ARIA-E, the subtype analysis shows that the risk of superficial siderosis (RR = 2.74) is significantly higher than that of microbleeds (RR = 1.97). This may be related to chronic vascular leakage induced by drugs: local inflammatory responses (such as complement activation and cytokine release) triggered during Aβ clearance can disrupt the integrity of the blood-brain barrier, leading to red blood cell extravasation. After chronic leakage, iron ions produced from the breakdown of hemoglobin exceed the brain’s clearance capacity (such as phagocytosis by glial cells or cerebrospinal fluid drainage), eventually depositing in the leptomeninges and brain surface. This mechanism is supported by the 2.63-fold increase in the risk of central nervous system superficial siderosis in this study (RR = 2.63, P < 0.0001). Although anti-Aβ treatment did not significantly increase the risk of conventional adverse events (such as falls and dizziness) (RR = 1.12, P = 0.02), the ARIA-related risk needs to be managed through the following strategies: adjusting monitoring frequency based on APOE ε4.

APOE genotype (particularly the ε4 homozygous status) may play a pivotal role in balancing the efficacy and safety of anti-Aβ monoclonal antibodies. Our study revealed that Alzheimer’s disease patients carrying the APOE ε4 homozygous allele exhibited significantly higher ARIA-E incidence following lecanemab/donanemab treatment compared to non-carriers, with a relatively attenuated risk increase observed in the lecanemab group. This phenomenon may be linked to the dose-dependent effects of the ε4 allele: ε4 homozygotes likely experience accelerated accumulation of monoclonal antibodies in brain parenchyma due to compromised blood-brain barrier integrity, thereby amplifying vascular edema risks triggered by Aβ-targeted clearance. These findings suggest that dissociation between biomarker response and clinical outcomes may be modulated by genotype. Consequently, we propose implementing a stratified management strategy for ε4 homozygous patients in clinical practice: pretreatment genetic screening could serve as a risk stratification tool, favoring gradual dose-escalation regimens (e.g., extended titration protocols for lecanemab) alongside intensified MRI monitoring (e.g., baseline scans followed by monthly evaluations during the first 3 months) to enable early ARIA detection (cite relevant imaging guidelines). However, current conclusions are constrained by the limited sample size of ε4 homozygotes (representing only X% of the included population). Future research should validate genotype-efficacy/safety causality and explore precision dose optimization through cross-trial individual patient data (IPD) meta-analyses.

In addition, aducanumab is also an effective drug for the treatment of AD. By comparison, it is suggested that the core features of Donanemab/Lecanemab, Aducanumab and symptomatic therapy: In terms of mechanism, Donanemab/Lecanemab targets amyloid β-protein (Aβ) fibrils and oligomers (such as TRAILBLAZER-ALZ two test) (Mintun et al., 2021), while Aducanumab mainly removes deposited plaques (EMERGE/ENGAGE subgroup analysis). Symptomatic therapy (such as acetylcholinesterase inhibitors) only regulates neurotransmitters; in terms of efficacy and safety, Donanemab showed that iADRS score delayed 35% cognitive decline in phase III trials (low/medium Tau subgroup was better) (Mintun et al., 2021), and Lecanemab (Clarity AD test) reduced CDR-SB by 27% (van Dyck et al., 2023), but both were accompanied by ARIA risk (edema 12.6%–17.3%). The incidence of ARIA in Aducanumab is higher (35% edema) and the efficacy is controversial. At the level of clinical applicability, Donanemab/Lecanemab requires strict screening of early AD patients (Aβ + and Tau below medium), high treatment costs (such as Lecanemab annual cost > USD 26,000) and reliance on biomarker monitoring (van Dyck et al., 2023). Although symptomatic therapy is more suitable for a wider population, it cannot delay disease progression. These differences highlight the importance of precise treatment and risk-benefit trade-offs in AD management.

The analysis in this study revealed significant differences in the safety profiles between lecanemab and donanemab, particularly demonstrating a higher ARIA-E risk in the donanemab group. This finding carries direct clinical implications: For patient populations who are APOE ε4 carriers or require rapid amyloid plaque clearance (e.g., rapidly progressive Alzheimer’s disease), clinicians should prioritize evaluating the short-term efficacy-risk balance of donanemab and consider increasing MRI monitoring frequency during pretreatment and early treatment phases (e.g., baseline and scans at months 3, 6, and 9) to dynamically manage ARIA events. These results highlight that drug selection should integrate patient preferences, anticipated treatment duration, and healthcare resource accessibility. For instance, lecanemab’s gradual dose-escalation regimen may be preferable for older adults with heightened sensitivity to tolerability, whereas donanemab might better align with clinical trial objectives requiring rapid biomarker improvements in the short term. Although the current sample size is limited by the predominance of Phase II studies, these findings provide a prospective framework for risk-stratified design in Phase III trials and real-world prescribing protocols. Future studies incorporating large-scale long-term follow-up data could further validate the generalizability of these clinical insights.

In summary, anti-Aβ monoclonal antibodies (Lecanemab/Donanemab) have demonstrated significant efficacy in improving cognitive function (as measured by the ADAS-Cog 14 scale) and activities of daily living (as assessed by the CDR-SB) in patients with early-stage Alzheimer’s disease, and have effectively reduced cerebral amyloid deposition (confirmed by PET imaging), thereby establishing their clinical value as disease-modifying treatments. However, the risk of amyloid-related imaging abnormalities (ARIA) associated with these treatments warrants heightened vigilance, particularly the elevated risk of ARIA-E in APOE ε4 carriers, as well as the occurrence of vascular complications such as superficial siderosis. Based on the current evidence, it is recommended that in clinical practice, APOE ε4 genotyping and baseline imaging assessments be integrated to develop individualized dosing and monitoring plans. Future research should focus on expanding the population coverage (especially including Asian cohorts) and extending follow-up periods to clarify long-term safety.

## Data Availability

The original contributions presented in the study are included in the article/supplementary material, further inquiries can be directed to the corresponding author.
